# Sodium Salicylate Suppresses GABAergic Inhibitory Activity in Neurons of Rodent Dorsal Raphe Nucleus

**DOI:** 10.1371/journal.pone.0126956

**Published:** 2015-05-11

**Authors:** Yan Jin, Bin Luo, Yan-Yan Su, Xin-Xing Wang, Liang Chen, Ming Wang, Wei-Wen Wang, Lin Chen

**Affiliations:** 1 CAS Key Laboratory of Brain Function and Diseases, School of Life Sciences, University of Science and Technology of China, Hefei, 230027, China; 2 Department of Otolaryngology-Head and Neck Surgery, Anhui Provincial Hospital, Hefei, 230001, China; 3 Department of Anatomy, Anhui Medical University, Hefei, 230032, China; 4 Auditory Research Laboratory, University of Science and Technology of China, Hefei, 230027, China; 5 Key Laboratory of Mental Health, Institute of Psychology, Chinese Academy of Sciences, Beijing, 100101, China; University of Wurzburg, GERMANY

## Abstract

Sodium salicylate (NaSal), a tinnitus inducing agent, can activate serotonergic (5-HTergic) neurons in the dorsal raphe nucleus (DRN) and can increase serotonin (5-HT) level in the inferior colliculus and the auditory cortex in rodents. To explore the underlying neural mechanisms, we first examined effects of NaSal on neuronal intrinsic properties and the inhibitory synaptic transmissions in DRN slices of rats by using whole-cell patch-clamp technique. We found that NaSal hyperpolarized the resting membrane potential, decreased the input resistance, and suppressed spontaneous and current-evoked firing in GABAergic neurons, but not in 5-HTergic neurons. In addition, NaSal reduced GABAergic spontaneous and miniature inhibitory postsynaptic currents in 5-HTergic neurons. We next examined whether the observed depression of GABAergic activity would cause an increase in the excitability of 5-HTergic neurons using optogenetic technique in DRN slices of the transgenic mouse with channelrhodopsin-2 expressed in GABAergic neurons. When the GABAergic inhibition was enhanced by optical stimulation to GABAergic neurons in mouse DRN, NaSal significantly depolarized the resting membrane potential, increased the input resistance and increased current-evoked firing of 5-HTergic neurons. However, NaSal would fail to increase the excitability of 5-HTergic neurons when the GABAergic synaptic transmission was blocked by picrotoxin, a GABA receptor antagonist. Our results indicate that NaSal suppresses the GABAergic activities to raise the excitability of local 5-HTergic neural circuits in the DRN, which may contribute to the elevated 5-HT level by NaSal in the brain.

## Introduction

Serotonin or 5-hydroxytryptamine (5-HT) is a monoamine neurotransmitter which is primarily found in the gastrointestinal tract, platelets, and the central nervous systems (CNS) of animals and humans [1,2,3,4,5]. In the CNS, serotonergic (5-HTergic) neurons are distributed in various raphe nuclei in the brain stem, and about half of the total are positioned in the dorsal raphe nucleus (DRN), a bilateral, neurochemically heterogeneous nucleus, located in the ventral part of periaqueductal grey [6,7]. Approximately 50% of 5-HTergic innervation to forebrain structures originate from the DRN (50–60% of the 5-HTergic neurons in the human CNS) [8,9,10]. In addition, most auditory nuclei in the CNS receive 5-HTergic projections from the DRN. These nuclei include the dorsal cochlear nucleus [11], the superior olivary complex [12,13], the nuclei of the lateral lemniscus [14], the inferior colliculus [11,15], and the auditory cortex [16,17]. A large proportion of non-5-HTergic neurons in the DRN are GABAergic fast-firing interneurons that comprise a major cell group in the DRN [10,18,19] for regulating 5-HTergic output.

The 5-HTergic system has been implicated in the expression of normal behaviors and in diverse psychiatric disorders, such as depression and anxiety [20]. It has also been implicated in many neurological symptoms such as tinnitus, a phantom auditory sensation without stimulation by an external sound source. For example, clinical studies revealed a significant increase in serum 5-HT in tinnitus patients and a significant increase in proportion of participants with elevated 5-hydroxyindoleacetic acid (5-HIAA), a metabolite of 5-HT, in the tinnitus group [21,22]. In animal models with tinnitus induced by a high dose of sodium salicylate (NaSal), an active component of aspirin (acetylsalicylic acid), there is a significant increase in extracellular 5-HT level in the inferior colliculus (268 ± 27% of the baseline) and the auditory cortex (277 ± 24% of the baseline) [23]. In our previous study, NaSal was shown to suppress a 5-HT-induced increase in the frequency of GABAergic spontaneous inhibitory postsynaptic currents (sIPSCs) in the inferior colliculus [24], suggesting that the modulatory function of 5-HT on the GABAergic neurons is altered in NaSal-induced tinnitus. In rats with NaSal-induced tinnitus, the perception of tinnitus is exacerbated after treatment of the 5-HT_2C_ receptor agonist *m*-chlorophenylpiperazine [25].

The increased 5-HT level in the brain of animal models with NaSal-induced tinnitus [23] suggests an increased 5-HTergic activity in the DRN. Indeed, neurons in the DRN of gerbils are activated after injection of NaSal, as indicated by the *c-fos* immunohistochemical marker [26]. These activated neurons were proven to be 5-HTergic neurons in a recent experiment [27]. The present study aimed to explore the neural mechanisms underlying the increased 5-HT level by NaSal using whole-cell patch-clamp recording and optogenetic techniques in brain slices. Our data show that NaSal raises the excitability of local 5-HTergic neural circuits by preferentially targeting GABAergic neurons in the DRN, which may contribute to the raised 5-HT level by NaSal in the brain of rodent animals.

## Materials and Methods

### Animals

Wistar rats (P13–P18, male or female) and the vesicular GABA transporter-channelrhodopsin-2-EYFP (VGAT-ChR2-EYFP) transgenic mice (hereinafter referred to as ChR2 transgenic mice, four–six week-old, male or female) were used in the present study. The rats were purchased from Shanghai SLAC Laboratory Animal Co. Ltd, China, and the ChR2 transgenic mice were donated by Dr. Guoping Feng (Massachusetts Institute of Technology, USA). The use of animals and the experimental procedures were approved by the Institutional Animal Care and Use Committee of University of Science and Technology of China. All efforts were made to minimize the number of animals used and their suffering.

### Acute brain slice preparation for electrophysiology

**Fig 1:**
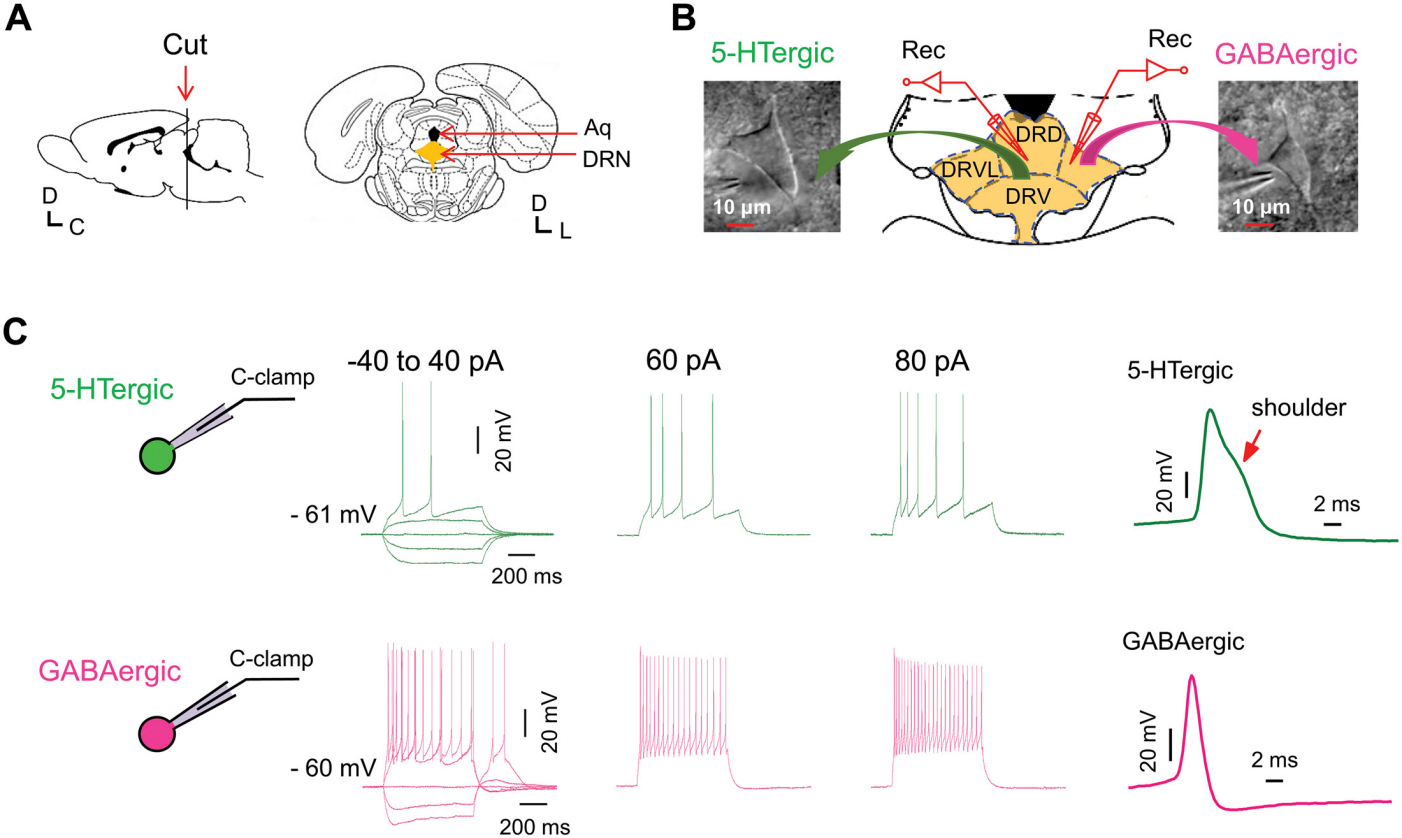
(A) Schematic showing coronal section of brain slices with location of the dorsal raphe nucleus (DRN) indicated. Aq, the cerebral aqueduct. C, caudal; D, dorsal; L, lateral. (B) A typical 5-HTergic neuron and a typical GABAergic neuron approached by recording micropipettes that were viewed under an upright microscope equipped with an infrared camera. DRD, the dorsal part of the DRN; DRV, the ventral part of the DRN; DRVL, the ventrolateral part of the DRN. (C) Voltage responses to current steps (duration, 700 ms; range: -40 to 80 pA, 20 pA/step) recorded from a 5-HTergic neuron (upper panels) and from a GABAergic neuron (lower panels). Note the difference in firing pattern and in action potential morphology between the two types of neurons.

The animal was decapitated, and the brain block containing the DRN was then rapidly removed. Before decapitation, the ChR2 transgenic mouse was deeply anesthetized by intra-peritoneal injection of pentobarbital sodium (0.5%, 15 μL/g) and then trans-cardially perfused with 25–30 mL of protective N-methyl-D-glucamine artificial cerebrospinal fluid (NMDG ACSF). Four or five coronal midbrain brain slices (250 μm for the rat and 200 μm for the ChR2 transgenic mouse) were cut (Fig 1A) with a vibrating microslicer (VT1200s, Leica, Gemany) in ice-cold ACSF (standard ACSF for the rat and NMDG ACSF for the ChR2 transgenic mouse). The cerebral aqueduct (Aq) of the midbrain was used as a landmark for locating the DRN [10]. During the slice preparation, the ACSF was bubbled with a mixture of oxygen and carbon dioxide gases (95% O_2_, 5% CO_2_) continuously. Brain slices from the rat were incubated in standard ACSF for at least 1 hour at 28°C. Brain slices from the ChR2 transgenic mouse were initially incubated in NMDG ACSF for 10–12 min at 33°C and then in N-2-hydroxyethylpiperaxine-N-2-ethanesulfonic acid (HEPES) ACSF for at least 1 hour at 28°C. The brain slice was transferred into a slice chamber (Warner Instruments, USA) for electrophysiological recording, and continuously perfused with aerated standard ACSF at 2.5–3 ml/min at 28°C maintained by an in-line solution heater (TC-344B, Warner Instruments, USA). Only one cell was recorded per brain slice.

The standard ACSF contained (in mM): 124 NaCl, 5 KCl, 2.4 CaCl_2_, 1.3 MgSO_4_, 1.2 KH_2_PO_4_, 26.2 NaHCO_3_ and 10 glucose (pH: 7.4, osmolarity: 300–305 mOsm/kg). The NMDG ACSF contained (in mM): 93 N-methyl-D-glucamine (NMDG), 2.5 KCl, 1.2 NaH_2_PO_4_, 30 NaHCO_3_, 20 HEPES, 25 glucose, 2 thiourea, 5 Na-ascorbate, 3 Na-pyruvate, 0.5 CaCl_2_, and 10 MgSO_4_, 3 glutathione (GSH) (osmolarity: 300–305 mOsm/kg). The pH of NMDG ACSF was titrated to 7.3–7.4 with concentrated HCl (which provides Cl^-^ counter-ions for NMDG). The HEPES ACSF contained (in mM): 92 NaCl, 2.5 KCl, 1.2 NaH_2_PO_4_, 30 NaHCO_3_, 20 HEPES, 25 glucose, 2 thiourea, 5 Na-ascorbate, 3 Na-pyruvate, 2 CaCl_2_, and 2 MgSO_4_, 3 glutathione (GSH) (pH: 7.4, osmolarity: 300–305 mOsm/kg).

### Whole-cell patch-clamp recording

The neurons in the DRN were visualized using a 40× water immersion objective on an upright microscope (FN1, Nikon, Japan) equipped with an interference contrast (IR/DIC) and infrared camera connected to the video monitor. Patch pipettes were pulled from glass capillaries with an outer diameter of 1.5 mm on a two-stage vertical puller (PC-10; Narishige, Tokyo, Japan). The resistance of the recording electrode filled with pipette solution was 3–5 MΩ for recordings from neurons in rat DRN and 7–8 MΩ for recordings from neurons in DRN slices of the ChR2 transgenic mouse. The signals were recorded by using Pulse software version 8.80 (HEKA Electronic, Germany) and PatchMaster software version 2.53 (HEKA Electronic, Germany) in a whole cell recording mode, filtered at 2.9 kHz and digitized at 10 kHz by using a HEKA EPC9 amplifier (HEKA Electronics, Germany) and a built-in PCI-16 interface board.

For recording the intrinsic membrane properties and the membrane currents, the pipettes were filled with intracellular solution containing (in mM): 130 K-gluconate, 2 MgCl_2_, 5 KCl, 0.6 EGTA, 10 HEPES, 2 Mg-ATP and 0.3 Na-GTP with osmolarity adjusted to 285–290 mOsm/kg and pH adjusted to 7.2 with KOH. For recording IPSCs, the pipettes were filled with intracellular solution containing (in mM): 120 KCl, 30 NaCl, 5 EGTA, 10 HEPES, 1 MgCl_2_, 0.5 CaCl_2_ and 2 Mg-ATP with osmolarity adjusted to 285–290 mOsm/kg and pH adjusted to 7.2 with KOH.

Spontaneous firing, current-evoked firing, the resting membrane potential and input resistance were recorded by using a current-clamp mode (I = 0 pA). A current pulse of -100 pA was used to monitor changes in membrane resistance following each sweep during recording the spontaneous firing. Data of current-evoked firing were only collected from neurons with a resting membrane potential more negative than -50 mV and an action potential amplitude that surpassed 0–5 mV. The recordings were made at least 5 min after establishing a whole cell configuration with a stable resting membrane potential.

Neurons were held at -60 mV by using voltage clamp mode for recording IPSCs. Only those with series resistance <30 MΩ and input resistance >100 MΩ were used for recording. If the series resistance changed by more than 20%, the experimental recording from that neuron would be terminated immediately. Kynurenic acid (KYN) at 4 mM was added in the standard ACSF to eliminate excitatory components. During the course of recordings, a 10 mV hyperpolarizing pulse was used at the end of every four sweeps (every 20 s) to monitor the series resistance on line. When mIPSCs were recorded, 1 μM tetrodotoxin (TTX, purchased from Hebei Aquatic Science and Technology Development Company, China) was added to the bath solution to eliminate spontaneous action potentials through blocking the voltage-gated sodium channel. After stable baseline recordings were acquired, NaSal was normally administered for 7–8 min. The liquid junction potential was not corrected during all experiments. The series resistance was not compensated for but was periodically monitored.

### Optical stimulation to GABAergic neurons in mouse brain slice

In the ChR2 transgenic mouse we used, blue light-sensitive channelrhodopsin-2 was expressed in GABAergic neurons [28]. For optical activation of GABAergic neurons, blue laser light (473 nm) was delivered by using a laser with the output power >100 mW (Shanghai Fiblaser Technology Co., Ltd. China) through a optical fiber of 200 μm in diameter positioned 0.2 mm away from the surface of the ventrolateral part of the DRN (DRVL) in the brain slice. The delivery of the laser light was electrically triggered and its power level was controlled by the voltage output from HEKA EPC9 amplifier. The specific power level of laser stimulation was calibrated to 5.5 mW or 18.3 mW with a laser power meter (AniLab Software and Instruments Co., Ltd. China). A train of laser light pulses (1 ms pulse width, 10 Hz) or a continuous laser light lasting for 450 ms, 1 s, 2 s, 3 s or 6 s were used.

### Data analysis

The methods for data analysis of intrinsic membrane properties were similar to those described previously [29]. The current-voltage (*I-V*) curve, which was changes in the membrane potential as a function of intracellular injected currents (-30 to -80 pA or -10 to -60 pA, -10 pA/step), was plotted and its slope was derived from the linear range of the curve. The slope of the *I-V* curve was defined as the input resistance of the cell membrane. The adaptation ratio of action potentials in the present study was defined as a ratio of first inter-spike interval after onset of 500 ms current injection to that immediately before offset of the injection. The voltage threshold (*V*_thresh_) of the action potential was the most negative voltage that had to be achieved by the current injection for all-or-none firing to occur [30]. The threshold current for firing was defined as the minimum strength of current injection required to elicit at least one or two spikes and used as an indicator of the neuronal sensitivity to depolarization. The amplitude of an action potential was defined as the difference between the threshold and the peak voltage of the action potential. The IPSC data were filtered off-line at 2 kHz and the membrane current data were filtered off-line at 1 kHz. The threshold value for detecting the IPSC was not less than 5 pA. The events detected were visually inspected to avoid electrical artifacts. The amplitude and frequency of IPSCs recorded before, during or after NaSal exposure were defined as the average measures across a time window of 60 s.

Off-line data analysis was carried out by using Pulse software version 8.80 (HEKA Electronic, Germany), FitMaster software version 2.53 (HEKA Electronic, Germany), Clampfit software version 10.0 (Axon Instruments, Inc, USA) and MiniAnalysis software version 6.03 (Synaptosoft Inc, USA). For two factorial analysis, statistical significance was determined by using the two-way repeated measures analysis of variance (two-way RM-ANOVA). If there was a significant main effect or a significant interaction between the two factors, one-way RM-ANOVA with Bonferroni correction was used for pairwise comparisons. For one factorial analysis, statistical significance was determined for planned comparisons by using paired or unpaired Student’s *t*-test. *P* <0.05 was considered significant. Data are expressed as mean ± standard error of mean (SEM). Kolmogorov-Smirnov two-sample test (K-S test) was used to test significant difference for cumulative probabilities. All analyses were performed by using SPSS software version 13 (IBM, USA) and Origin Pro software version 8.0 (OriginLab Corporation, USA). The processed data were imported into Origin software version 8.0 for generating graphs.

### Drugs

NaSal was dissolved in standard ACSF just before use. The concentration of NaSal throughout our experiment was 1.4 mM, a typical concentration found in the cerebrospinal fluid of animal models with NaSal-induced tinnitus [31,32]. Stock solution of the picrotoxin (PTX, a GABA receptor antagonist) and (R)-(+)-8-Hydroxy-DPAT hydrobromide (8-OH-DPAT, a 5-HT_1A_ agonist) was made in dimethyl sulphoxide (DMSO) and other drug solution was made in ultrapure H_2_O. Drugs were dissolved to the final concentration in standard ACSF and applied by a peristaltic pump (Lange Pump, BT00-100M, China). Complete replacement of the medium in the chamber took 50–60 s. All the chemical compounds were purchased from Sigma-Aldrich, Inc., USA unless otherwise specified.

## Results

### Identification of 5-HTergic and GABAergic neurons in rat DRN

We positioned the electrode in the area of the dorsal and ventral subdivisions along the midline of the DRN (DRD and DRV) for recordings from 5-HTergic neurons and within the ventrolateral subdivision (DRVL) for those from GABAergic neurons (Fig 1B) [33,34,35]. Neuronal types (5-HTergic or GABAergic neurons) were identified based on their distinct morphological [19], electrophysiological [19,36,37,38,39,40] and pharmacological properties [41,42]. According to previous studies by others [19, 43], we initially considered the neuron with a large fusiform or multipolar cell body as a putative 5-HTergic neuron, and the neuron with a small cell body and aspiny dendrites as a putative GABAergic neuron (Fig 1B). The neuronal types were further confirmed by a set of electrophysiological and pharmacological criteria as detailed in Table 1. Sample action potentials recorded from indentified 5-HTergic and GABAergic neurons with distinct electrophysiological properties are shown in Fig 1C. The identified two types of neurons exhibited distinct differences in current-evoked firing (S1A Fig), inter-spike interval (S1 Fig), action potential morphology (S1C Fig), the time constant (tau) of current-voltage responses (S2 Fig), and pharmacological responses (S3 Fig).

**Table 1:** Electrophysiological and pharmacological criteria used for identification of 5-HTergic and GABAergic neurons in rat dorsal raphe nucleus (DRN).

Criterion	Measurements	5-HTergic	GABAergic
**Electrophysiological**	AP shoulder^1^	Yes	No
	Firing rate	Low	High
	Adaption ratio of firing	Low	High
	AP half width	Broad	Narrow
	Rebound firing	No	Yes
	Tau^2^	Long	Short
**Pharmacological**	5-HT induced current	Outward	Inward
	8-OH-DPAT induced current	Large, outward	Small, outward, or inward
	AP Firing	Decreased by 5-HT or blocked by 8-OH-DPAT	Increased by 5-HT

^1^Shoulder was a delayed repolarization on the single action potential.

^2^Tau was measured as the amount of time for the membrane to charge to 63% of maximum in response to a small hyperpolarizing current pulse (-30 to -50 pA).

AP, action potential; Tau, time constant; 8-OH-DPAT, (R)-(+)-8-Hydroxy-DPAT hydrobromide.

### NaSal hyperpolarized the resting membrane potential and decreased the membrane input resistance in GABAergic neurons, but not in 5-HTergic neurons, of rat DRN

**Fig 2:**
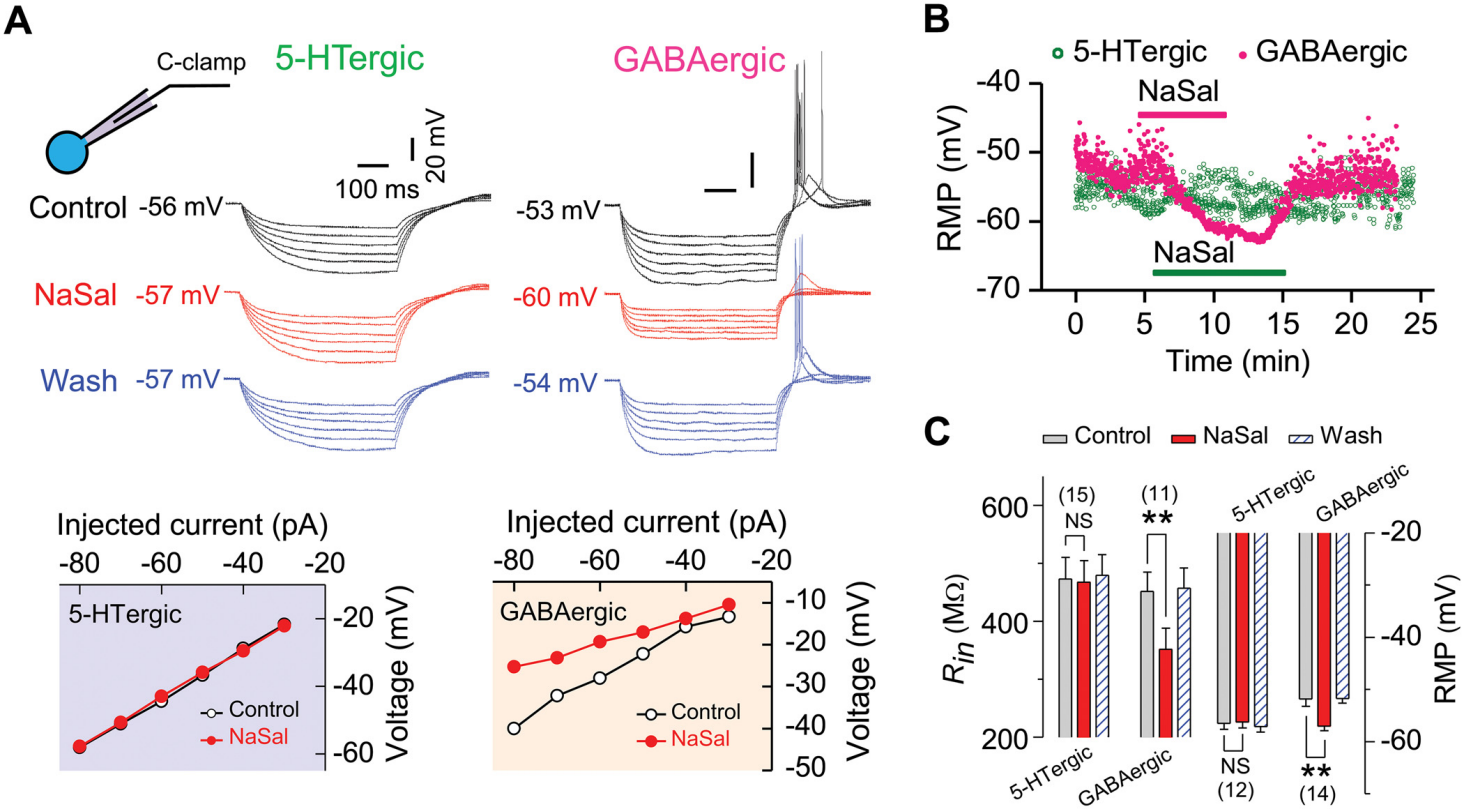
Sodium salicylate (NaSal) hyperpolarized the resting membrane potential (RMP) and reduced the input resistance (*R*_in_) in GABAergic, but not in 5-HTergic, neurons of rat DRN. (A) Sample traces of voltage responses to a series of hyperpolarizing current steps (-30 to -80 pA, -10 pA/step; duration: 500 ms) recorded from a 5-HTergic neuron (upper left panel) and from a GABAergic neuron (upper right panel) before (Control), during (NaSal) and after (Wash) NaSal. Voltage-current plots derived from these traces are shown in lower panels. (B) Time courses of the RMP recorded from the same neurons as in (A) in response to application of NaSal (solid horizontal bars). (C) Statistics showing a significant change in the *R*_in_ and the RMP following NaSal in GABAergic, but not in 5-HTergic, neurons. Sample sizes indicated inset. Vertical line bars represent one standard error. **P <0.01 and ^NS^ *P >*0.05 relative to control (two-way RM-ANOVA and one-way RM-ANOVA with Bonferroni correction, 3 pairwise comparisons).

Under the current clamp mode, application of 1.4 mM NaSal reversibly hyperpolarized the resting membrane potential in GABAergic neurons (-51.87 ± 1.41 vs. -57.00 ± 0.92 mV, n = 14, *P <*0.01) (Fig 2A, upper panels; Fig 2B and 2C), but not in 5-HTergic neurons (-56.50 ± 1.13 vs. -56.25 ± 1.17 mV, n = 12, *P >*0.05) (Fig 2A, upper panels; Fig 2B and 2C) of rat DRN. In addition, NaSal decreased the input resistance, which was calculated from the *I-V* plots (Fig 2A, lower panels), in GABAergic neurons (451.63 ± 33.06 vs. 351.27 ± 37.18 MΩ, n = 11, *P <*0.01, Fig 2C), but not in 5-HTergic neurons (473.12 ± 37.03 vs. 467.83 ± 36.63 MΩ, n = 15, *P >*0.05, Fig 2C). These results indicate that NaSal preferentially decreases the membrane excitability of GABAergic neurons, but not of 5-HTergic neurons, in rat DRN.

### NaSal decreased the spontaneous firing of GABAergic neurons, but not of 5-HTergic neurons, in the rat

**Fig 3:**
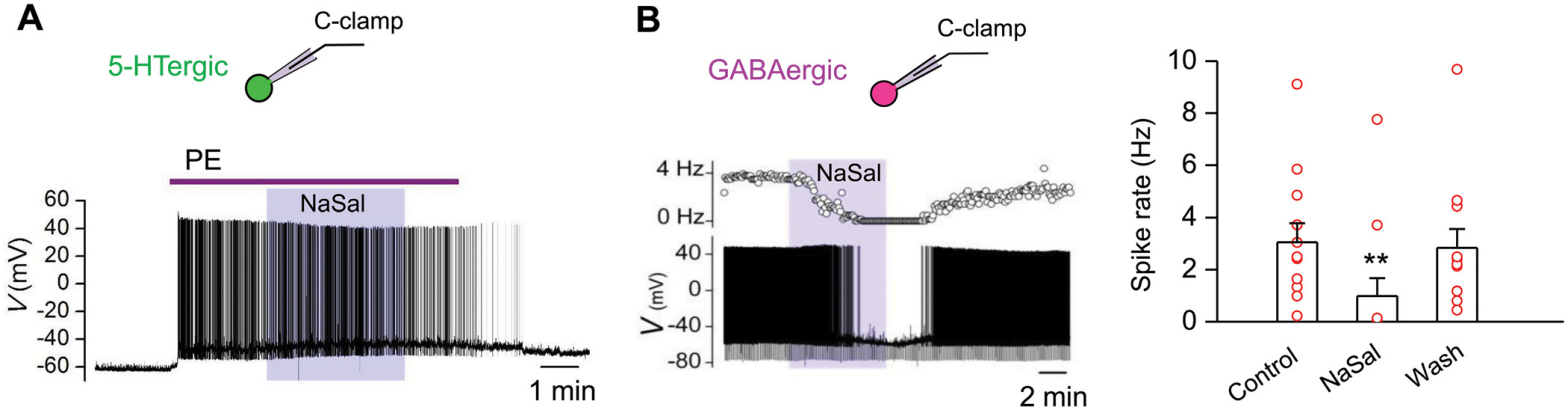
NaSal inhibited the spontaneous firing of GABAergic neurons in rat DRN. (A) NaSal had no effect on firing evoked by phenylephrine (PE, 3 μM) recorded from a 5-HTergic neuron. (B) NaSal suppressed spontaneous firing in GABAergic neurons (n = 12). Vertical line bars represent one standard error. **P <0.01 relative to control (one-way RM-ANOVA with Bonferroni correction, 3 pairwise comparisons). Downward deflections are voltage responses to intracellular injection of a -100 pA current pulse for monitoring changes in the membrane resistance.

The spontaneous firing activity of 5-HTergic neurons in the DRN is related to the tonically active noradrenergic system [38,44,45,46]. However, the noradrenergic inputs are severed and 5-HTergic neurons are often quiescent in brain slices [38,44]. In the present study, we found that the spontaneous firing activity of the 5-HTergic neuron gradually diminished within 5–6 min after onset of the recording (data not show). By adding 3 μM phenylephrine (PE, a α_1_-adrenoceptor agonist; purchased from Tocris Cookson Ltd., Bristol, UK), the spontaneous firing of 5-HTergic neurons was restored, and NaSal had no effect on the firing (Fig 3A). In contrast, the spontaneous firing activity of GABAergic neurons (12 out of 35 neurons) was high and sustainable (Fig 3B, left panel). NaSal significantly and reversibly decreased the spontaneous firing in GABAergic neurons from 3.05 ± 0.73 Hz to 0.98 ± 0.69 Hz (n = 12, *P <*0.01, Fig 3B, right panel). These results indicate that the NaSal suppresses the spontaneous activity of GABAergic neurons, but not that of 5-HTergic neurons, in rat DRN.

### NaSal depressed the current-evoked firing of GABAergic, but not 5-HTergic, neurons in rat DRN

**Fig 4:**
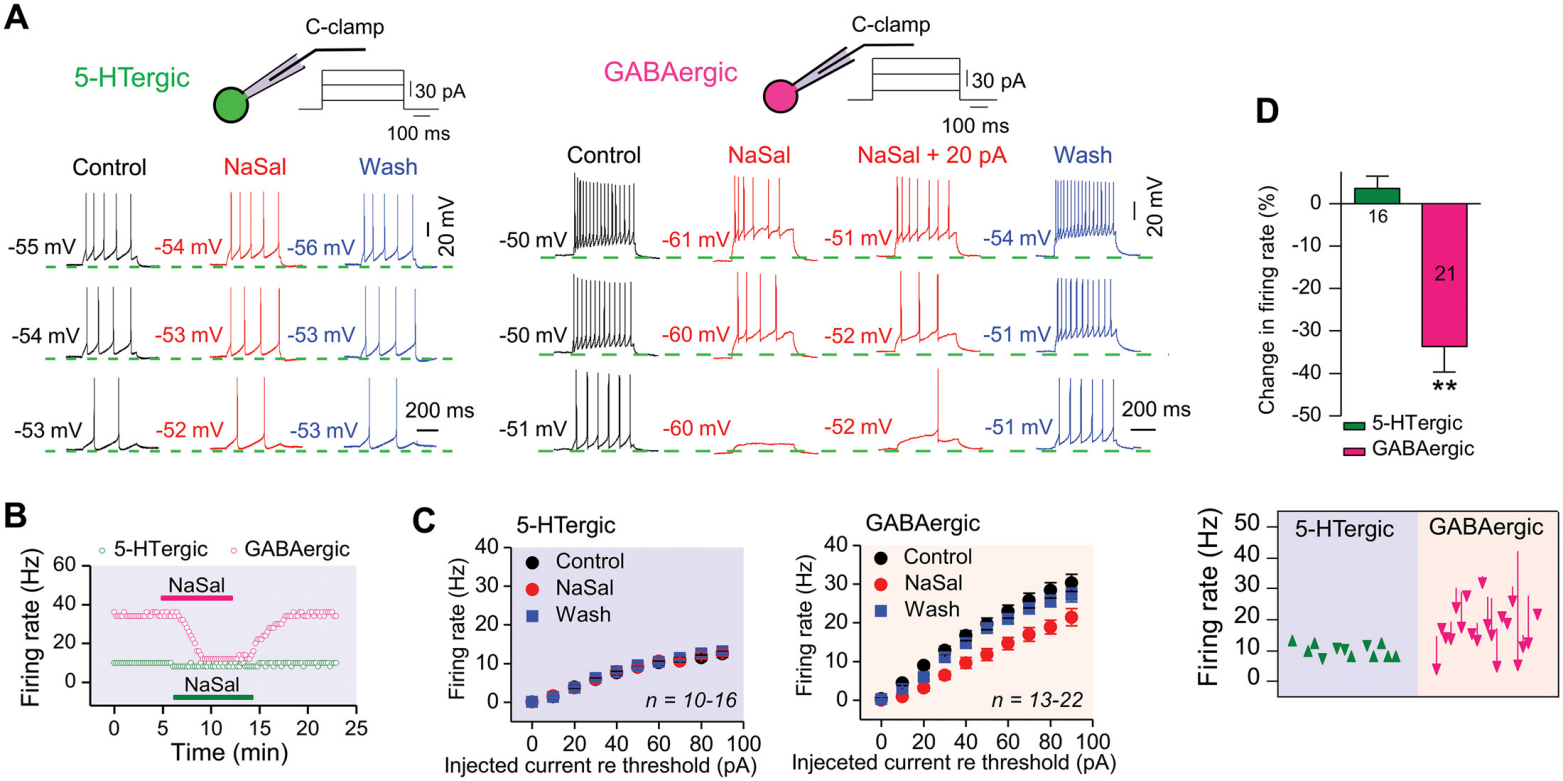
NaSal suppressed the current-evoked firing in GABAergic, but not in 5-HTergic, neurons of rat DRN. (A) Sample action potential trains in response to three depolarizing current steps (+20, 50, 80 pA; duration: 500 ms) in a 5-HTergic neuron and a GABAergic neuron following NaSal. The firing of the GABAergic neuron remained depressed by NaSal even its hyperpolarized membrane potential was adjusted back to the control level with a 20 pA current injection. RMPs indicated beside the traces. (B) Time courses of changes in firing rate of the same neurons in response to a +80 pA depolarizing current during NaSal application. (C) NaSal significantly suppressed input-output functions of current-evoked firing in GABAergic (*P* <0.01 relative to control, two-way RM-ANOVA), but not in 5-HTergic, neurons (*P* >0.05 relative to control, two-way RM-ANOVA). (D) Bar graph (upper panel) and trajectory plots (bottom panel) showing changes in firing rate of 5-HTergic and GABAergic neurons in response to a 60 pA depolarizing current re threshold following application of NaSal (two-way RM-ANOVA and one-way RM-ANOVA with Bonferroni correction). Sample sizes are indicated in inset. **P <0.01. Vertical line bars represent one standard error.

Effects of NaSal on the current-evoked spikes were tested by injecting 3 depolarizing current steps (20, 50, 80 pA for 500 ms) into neurons. For 5-HTergic neurons, NaSal had little or no effect on the rate of current-evoked firing (Fig 4A, left panel) (3.57 ± 2.87%, n = 16, *P >*0.05, Fig 4D). For GABAergic neurons, however, the rate of current-evoked firing was dramatically decreased after NaSal application (Fig 4A, right panel) (-33.68 ± 6.03%, n = 21, *P <*0.01, Fig 4D), even after the hyperpolarized membrane potential was compensated for by injecting a +20 pA current (Fig 4A, right panel). The time courses of changes in firing rate of a 5-HTergic neuron and that of a GABAergic neuron in response to a +80 pA depolarizing current during NaSal application are shown in Fig 4B.

Effects of NaSal on the rate of neuronal firing evoked by depolarizing currents with a broad range of strengths (from 0 to 90 pA re-threshold for 500 ms, 10 pA/step) was examined (Fig 4C and S4 Fig). NaSal had no significant effect on the input-output function of firing rate in 5-HTergic neurons (n = 10–16, *P >*0.05, Fig 4C, left panel), but significantly suppressed that in GABAergic neurons (n = 13–22, *P <*0.01, Fig 4C, right panel).

NaSal also affected the action potential properties of GABAergic neurons, but not those of 5-HTergic neurons (Table 2). In GABAergic neurons, NaSal increased the voltage threshold (*V*_thresh_) of the action potential (S5A Fig) and the corresponding threshold current (S5B Fig), and reduced the amplitude, rise slope and decay slope of the action potential (Table 2).

**Table 2:** Effects of 1.4 mM sodium salicylate (NaSal) on the action potential (AP) of 5-HTergic and GABAergic neurons in rat DRN.

Measurements	5-HTergic neuron (n = 16)		GABAergic neuron (n = 14)	
	Before NaSal	After NaSal	Before NaSal	After NaSal
**AP threshold (mV)**	-32.83 ± 0.89	-32.48± 0.83	-32.41 ± 0.83	-31.21 ± 0.95*
**AP amplitude (mV)**	80.82 ± 1.88	79.77 ± 1.79	73.88 ± 2.22	66.74 ± 2.60**
**AHP amplitude (mV)**	-23.44 ± 0.57	-23.53 ± 0.59	-24.44 ± 1.29	-26.79 ± 1.28
**Half-width (ms)**	1.51 ± 0.08	1.52 ± 0.08	0.91 ± 0.05	0.93 ± 0.05
**Rise slope (mV/ms)**	140.86 ± 10.81	137.97 ± 11.96	143.71 ± 13.22	129.36 ± 14.09*
**Decay slope (mV/ms)**	-34.23 ± 1.42	-34.23 ± 1.63	-78.02 ± 5.93	-71.66 ± 5.50*

Data are expressed as mean ± SEM. Asterisks * and ** indicate *P <*0.05 and *P <*0.01 relative before NaSal, respectively (two-way ANOVA and paired Student’s *t*-test). AHP, afterhyperpolarization.

### NaSal suppressed the GABAergic IPSCs in 5-HTergic neurons of rat DRN

**Fig 5:**
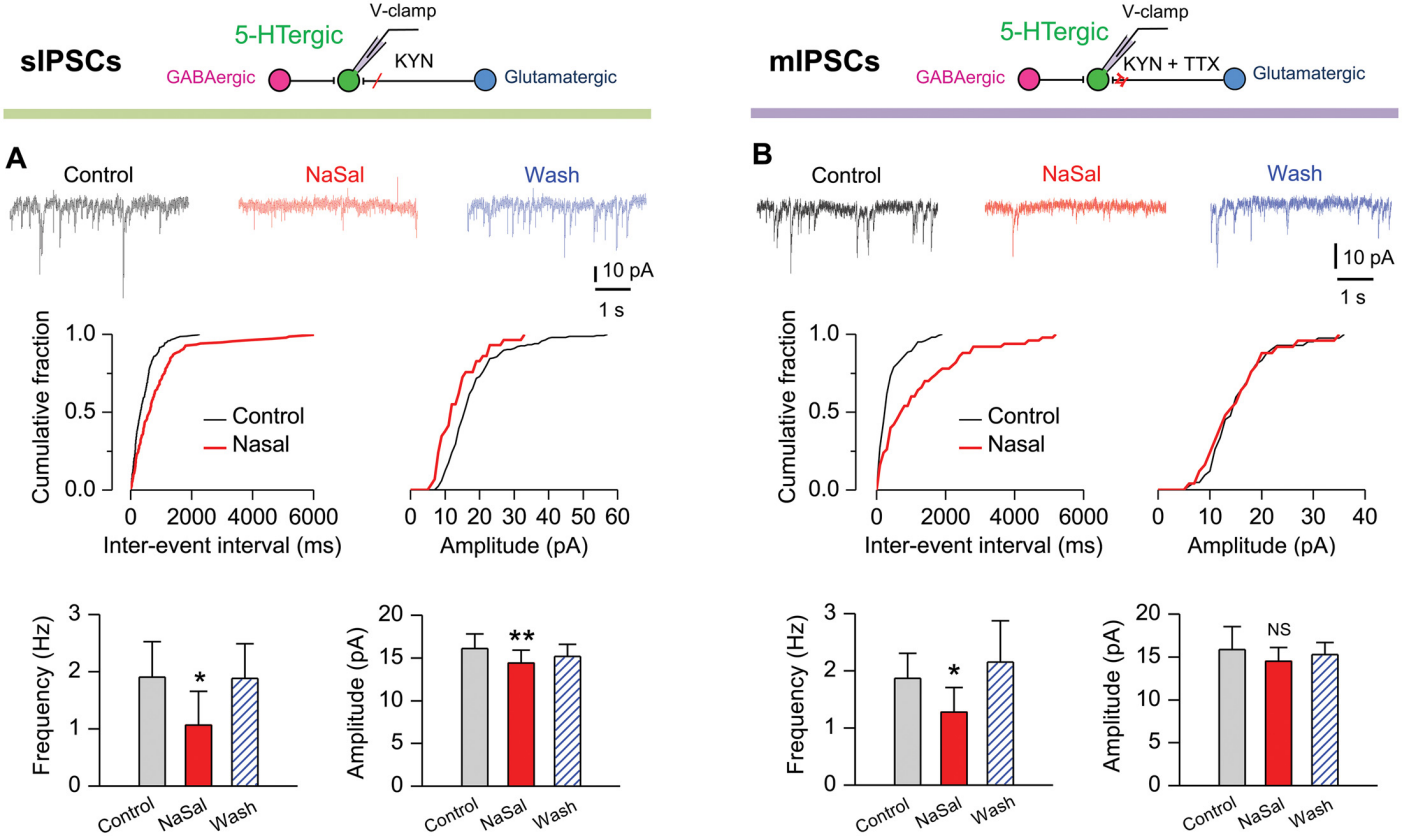
NaSal suppressed the spontaneous and miniature inhibitory postsynaptic currents (sIPSCs and mIPSCs) significantly in 5-HTergic neurons of rat DRN. Representative IPSC traces, cumulative probability graphs and statistics showing that NaSal decreased the frequency and the amplitude of sIPSCs (n = 8) (A) and mIPSCs (n = 12) (B) in 5-HTergic neurons. Vertical line bars represent one standard error. *P <0.05 and **P <0.01 and ^NS^ *P* > 0.05 relative to control (paired Student’s *t*-test).

GABAergic spontaneous (action potential-dependent) inhibitory postsynaptic currents (sIPSCs) in DRN neurons were recorded with 4 mM KYN added to block the excitatory synaptic transmission and with the neurons voltage-clamped at -60 mV. Application of 10 μM bicuculline (BIC), completely eliminated sIPSCs of 5-HTergic neurons, confirming that these events are mediated by GABA_A_ receptors (S6 Fig). Cumulative fraction plots and group data (n = 8) show that NaSal significantly reduced the frequency (K-S test, *P <*0.001; paired Student *t*-test, *P* <0.05) and amplitude (K-S test, *P <*0.001; paired Student *t*-test, *P* <0.01) of sIPSCs in 5-HTergic neurons (Fig 5A).

Miniature inhibitory postsynaptic currents (mIPSCs) in 5-HTergic neurons were recorded with 1 μM TTX added to block the fast sodium channel. Cumulative fraction plots and group data (n = 12) show that NaSal significantly depressed the frequency (K-S test, *P <*0.001; paired Student *t*-test, *P* <0.05), but not the amplitude (K-S test, *P >*0.05; paired Student *t*-test, *P* >0.05), of mIPSCs in 5-HTergic neurons (Fig 5B). Effects of NaSal on sIPSCs and mIPSCs are summarized in Table 3. These results suggest that NaSal reduces the GABAergic synaptic transmission to 5-HTergic neurons mainly through a presynaptic mechanism in rat DRN.

**Table 3:** Effects of NaSal on the GABAergic synaptic transmissions to 5-HTergic neurons of rat DRN.

Measurements	Frequency (Hz)		Amplitude (pA)	
	Before NaSal	After NaSal	Before NaSal	After NaSal
**sIPSC (n = 8**)	1.90 ± 0.62	1.07 ± 0.59**	16.10 ± 1.70	14.39 ± 1.54**
**mIPSC (n = 12)**	1.87 ± 0.44	1.28 ± 0.51*	15.82 ± 2.68	14.05 ± 1.61

Data are expressed as mean ± SEM. Asterisks * and ** indicate *P <*0.05 and *P <*0.01 relative to those before NaSal, respectively (paired Student’s *t*-test).

### NaSal increased current-evoked firing of 5-HTergic neurons in the DRN of the ChR2 transgenic mouse

**Fig 6:**
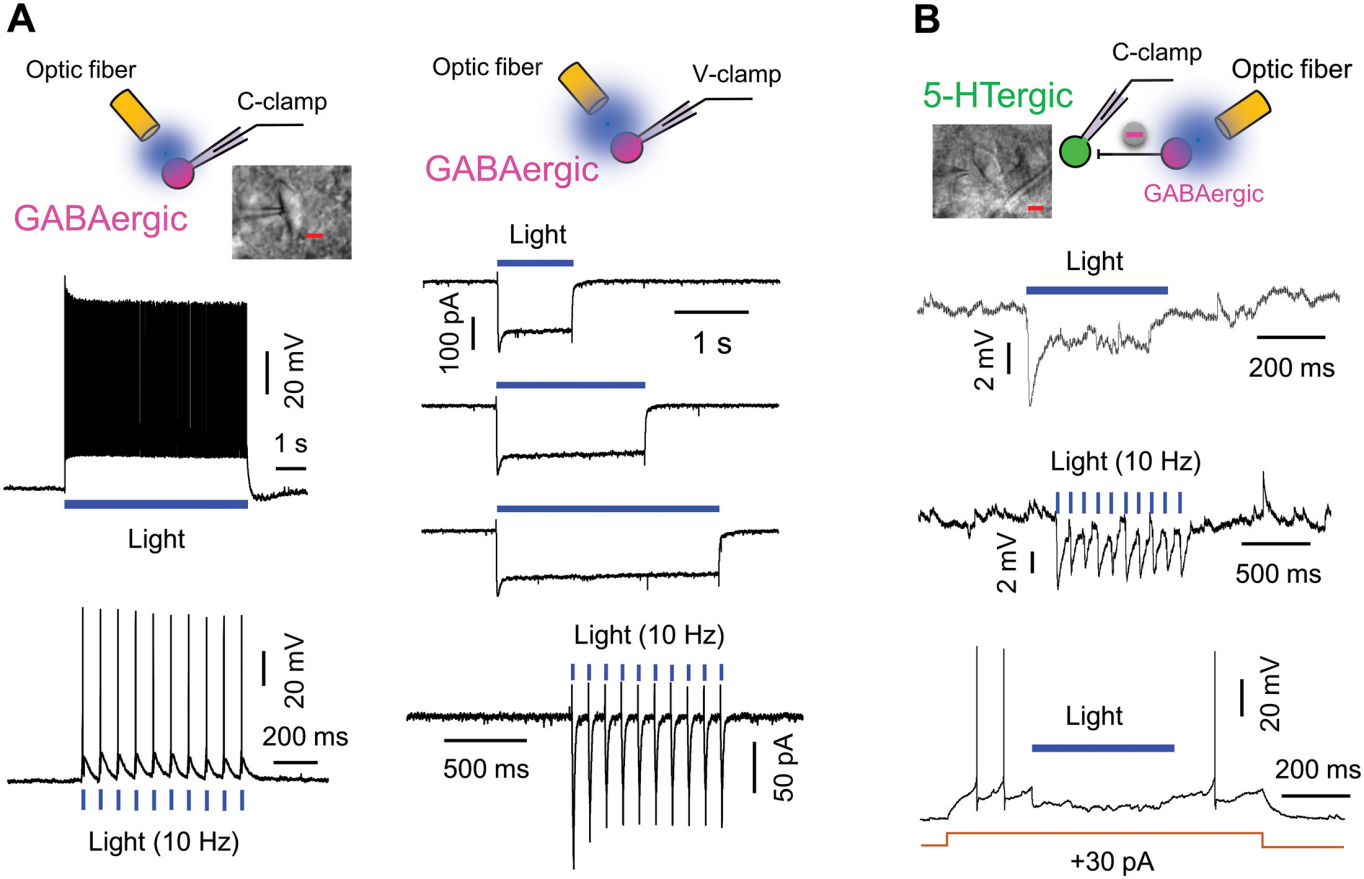
Optical stimulation excited GABAergic neurons to inhibit 5-HTergic neurons in the DRN of the ChR2 transgenic mouse (VGAT-ChR2-EYFP transgenic mouse). (A) Sample recordings of neuronal firing (left panels) and inward photocurrents (right panels) from a GABAergic neuron in response to a constant or a 10 Hz pulse (1 ms pulse width) laser light (5.5 mW, 473 nm). (B) Sample recordings showing the hyperpolarized RMP (upper and middle panels) and suppressed current-evoked firing (lower panel) in a 5-HTergic neuron by a constant or a 10 Hz pulse (1 ms pulse width) laser light (18.3 mW, 473 nm). High magnification IR/DIC image of a representative GABAergic neuron and a representative 5-HTergic neuron are shown. Scale bar: 10 μm.

NaSal suppressed GABAergic activity but failed to raise the excitability of 5-HTergic neurons in DRN slices of the rat (Figs 2–4), which may be accounted for by a low baseline of the GABAergic inhibition under an *in vitro* condition. Thus, we examined effects of NaSal on the excitability of 5-HTergic neurons with enhanced GABAergic inhibition by using optical stimulation to DRN slices of the ChR2 transgenic mouse. A blue light excited GABAergic neurons (Fig 6A) and strongly inhibited 5-HTergic neurons (Fig 6B), indicating that optical stimulation enhances the GABAergic inhibition in DRN slices of the ChR2 transgenic mouse.

**Fig 7:**
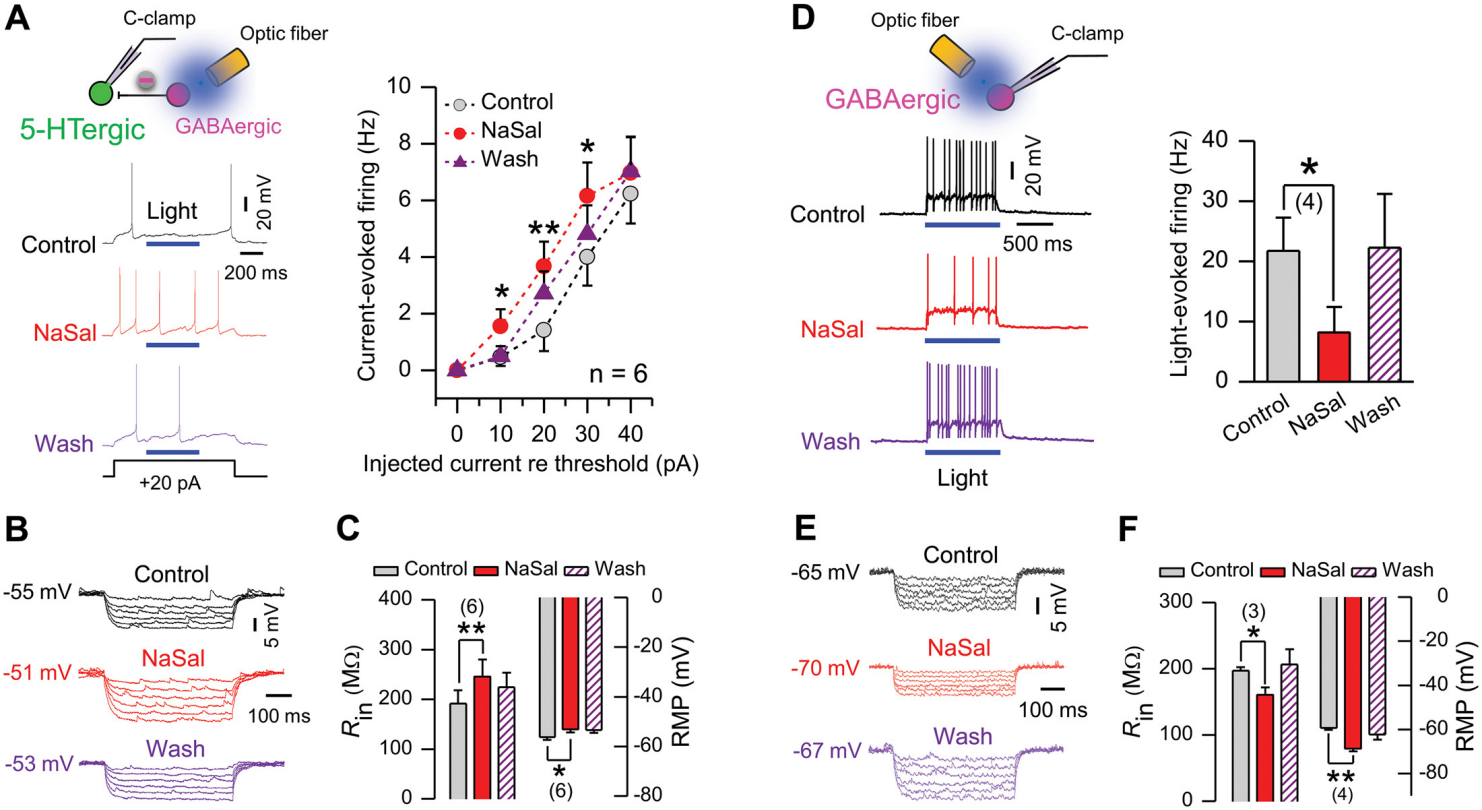
NaSal increased the excitability of 5-HTergic neurons, but decreased that of GABAergic neurons in the DRN of the ChR2 transgenic mouse. (A) Sample traces (left panel) and group data (right panel, two RM-ANOVA and paired Student’s *t*-test) showing that NaSal increased the current-evoked firing of 5-HTergic neurons with enhanced GABAergic inhibition by optical stimulation (450 ms, 18.3 mW, 473 nm). (B) Sample recordings from a 5-HTergic neuron showing that NaSal increased voltage responses to a series of hyperpolarizing currents (-10 to -60 pA, -10 pA/step, duration: 500 ms). The RMPs are indicated. (C) Group data showing that NaSal depolarized the RMP and increased the *R*_in_ in 5-HTergic neurons (paired Student’s *t*-test). (D) Sample traces (left panel) and group data (right panel, paired Student’s *t*-test) showing that NaSal suppressed the firing of GABAergic neurons evoked by laser light (1 s, 5.5 mW, 473 nm). (E) Sample recordings from a GABAergic neuron showing that NaSal decreased voltage responses to a series of hyperpolarizing currents (-10 to -60 pA, -10 pA/step, duration: 500 ms). The RMPs are indicated. (F) Group data showing that NaSal hyperpolarized the RMP and decreased the *R*_in_ in GABAergic neurons (paired Student’s *t*-test). Sample sizes are indicated in inset. Vertical line bars represent one standard error. *P <0.05 and **P <0.01 relative to control.

In 5-HTergic neurons, perfusion of NaSal plus blue light stimulation to the DRN significantly increased the current-evoked firing (n = 6, *P <*0.05 or 0.01, Fig 7A). Meanwhile, NaSal increased the input resistance (191.38 ± 26.66 vs. 245.29 ± 34.88 MΩ, n = 6, *P <*0.01, Fig 7B and 7C) and depolarized the resting membrane potential (-56.19 ± 1.23 vs. -53.09 ± 1.28 mV, n = 6, *P <*0.05, Fig 7B and 7C). These results indicate that NaSal increases the excitability of 5-HTergic neurons *in vitro* when the floor of the GABAergic inhibition is raised by optical stimulation to the DRN of the ChR2 transgenic mouse.

**Fig 8:**
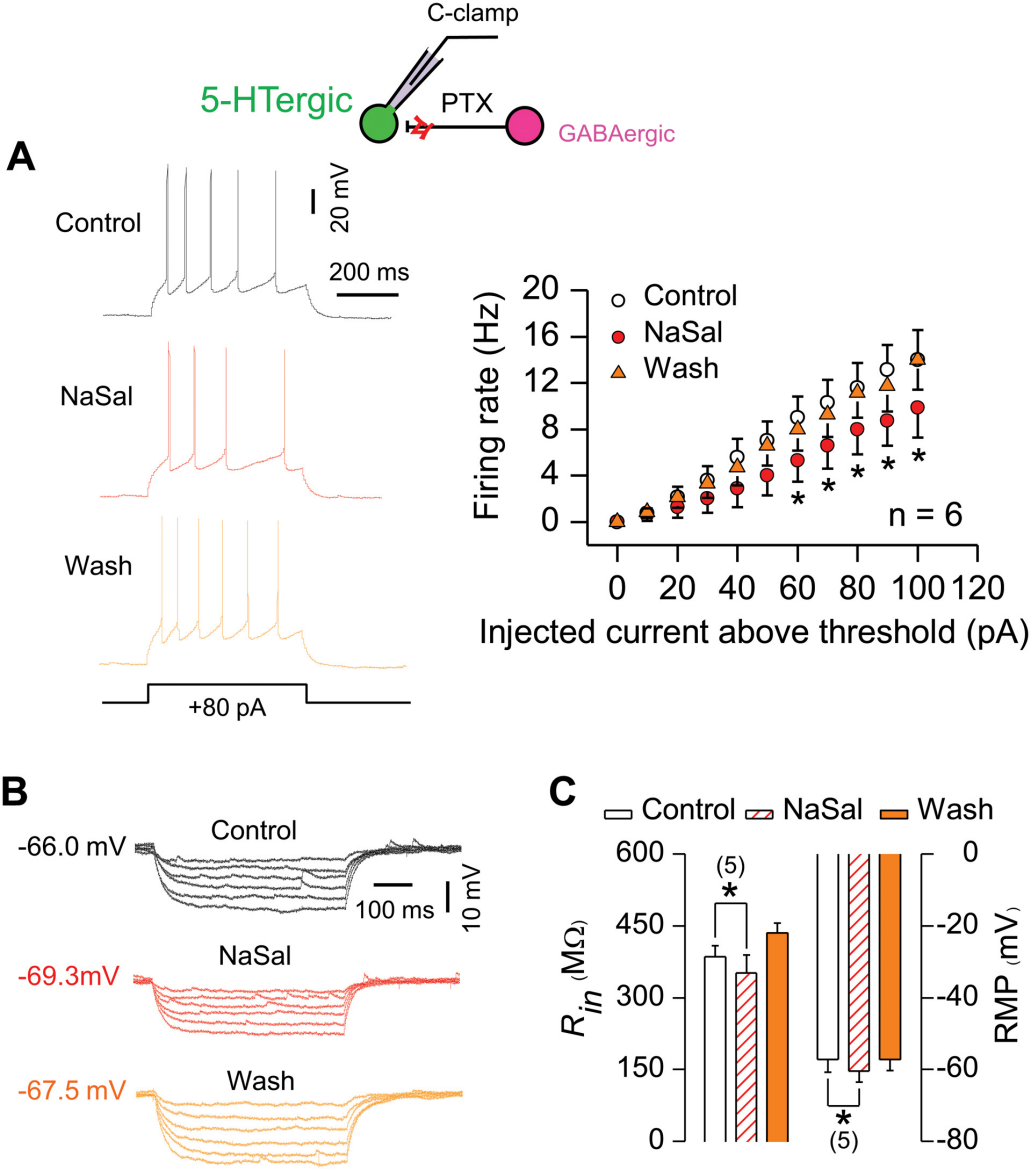
NaSal failed to increase excitability of 5-HTergic neurons in the absence of the GABAergic inhibition in the DRN of the ChR2 transgenic mouse. (A) Sample recordings (left panel) and group data (right panel, two RM-ANOVA and paired Student’s *t*-test) showing that NaSal did not increase the current-evoked firing of 5-HTergic neurons with the GABAergic synaptic transmission blocked by 100 μM PTX. (B) Sample recordings from a 5-HTergic neuron showing that NaSal decreased voltage responses to a series of hyperpolarizing currents (-10 to -60 pA, -10 pA/step, duration: 500 ms). The RMPs are indicated. (C) Group data showing that NaSal hyperpolarized the RMP and decreased the *R*_in_ in 5-HTergic neurons (paired Student’s *t*-test). Sample sizes are indicated in inset. Vertical line bars represent one standard error. *P <0.05 relative to control.

In GABAergic neurons, NaSal significantly depressed light-evoked firing (21.71 ± 5.59 vs. 8.16 ± 4.23 Hz, n = 4, *P <*0.05, Fig 7D). Meanwhile, NaSal decreased the input resistance (197.21 ± 5.30 vs. 160.64 ± 10.84 MΩ, n = 3, *P <*0.05, Fig 7E and 7F) and hyperpolarized the resting membrane potential (-59.32 ± 0.84 vs. -68.62 ± 1.29 mV, n = 5, *P <*0.01, Fig 7E and 7F). These results indicate that NaSal depresses the GABAergic inhibition in the DRN of the ChR2 transgenic mouse. When the GABAergic inhibition was blocked by 100 μM PTX, a GABA receptor antagonist, NaSal was no longer able to increase the excitability of 5-HTergic neurons (Fig 8), indicating that the depressed GABAergic activity by NaSal accounts for the increased excitability of 5-HTergic neurons.

### NaSal suppressed laser light-enhanced sIPSCs in 5-HTergic neurons in the DRN of the ChR2 transgenic mouse

**Fig 9:**
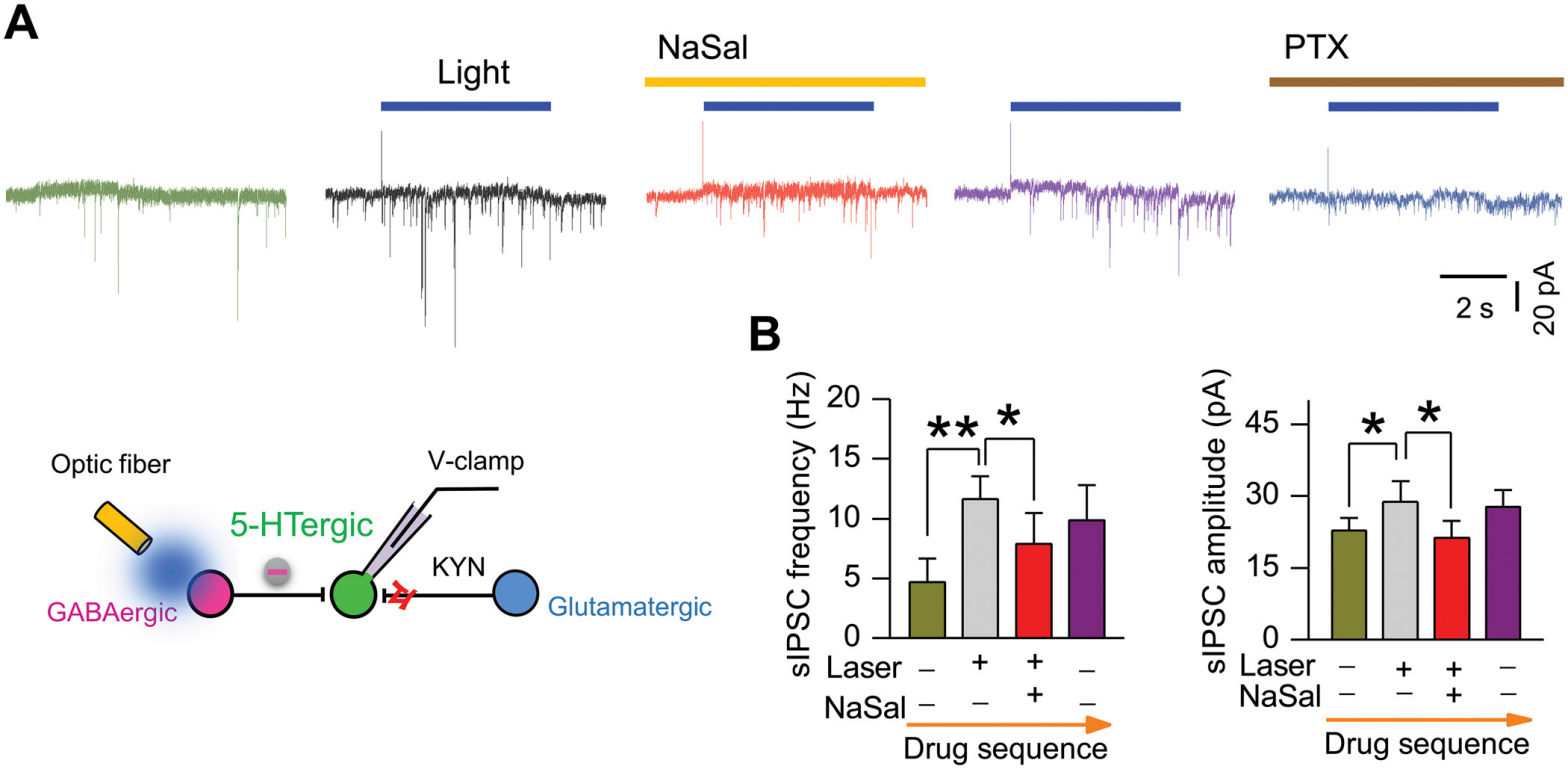
NaSal suppressed laser light-enhanced sIPSCs in 5-HTergic neurons in the DRN of the ChR2 transgenic mouse. (A) Sample traces of laser light-enhanced sIPSCs recorded from an identified 5-HTergic neuron in the absence and the presence of NaSal. Note that 100 μM PTX could block the sIPSCs. (B) Group data showing that NaSal suppressed both frequency (left panel) and amplitude (right panel) of laser light-enhanced sIPSCs in 5-HTergic neurons (n = 5, paired Student’s *t*-test). Laser light: 6 s, 18.3 mW, 473 nm. Vertical line bars represent one standard error. **P <0.01, *P <0.05.

Finally, we examined effects of NaSal on the inhibitory synaptic transmissions to 5-HTergic neurons in the DRN of the ChR2 transgenic mouse. Fig 9A shows raw traces of sIPSCs recorded from an identified 5-HTergic neuron under the influence of light stimulation and NaSal. Optica stimulation to the DRN boosted the sIPSC frequency from 4.10 ± 1.96 Hz to 11.64 ± 1.90 Hz (n = 5, *P <*0.01, Fig 9B, left panel) and increased the sIPSC amplitude from 22.69 ± 2.74 pA to 28.77 ± 4.38 pA (n = 5, *P <*0.05, Fig 9B, right panel) in 5-HTergic neurons. Application of NaSal during light stimulation reversibly suppressed the frequency and amplitude of sIPSCs back to 7.87 ± 2.57 Hz and 21.17 ± 3.65 pA, respectively (n = 5, *P <*0.05, Fig 9B). The recorded sIPSCs could be eliminated by 100 μM PTX (Fig 9A), a GABA receptor antagonist. These results indicate that NaSal can reduce the GABAergic inhibitory synaptic transmissions to 5-HTergic neurons in the DRN of the ChR2 transgenic mouse (Fig 9), as it can in rat DRN (Fig 5).

## Discussion

In the present study, we demonstrate that NaSal can suppress the GABAergic inhibitory activities to raise the excitability of local 5-HTergic neural circuits in the rodent DRN *in vitro*. Evidence supporting this conclusion includes: (1) NaSal decreased the excitability of GABAergic neurons (Figs 2–4 and 7, Table 2) and suppressed the GABAergic inhibitory synaptic transmissions to 5-HTergic neurons (Figs 5 and 9; Table 3); (2) NaSal increased the excitability of 5-HTergic neurons when the GABAergic inhibition was enhanced by optical stimulation (Fig 7); (3) NaSal was no longer able to increase the excitability of 5-HTergic neurons when the GABAergic inhibitory synaptic transmission was blocked by PTX, a GABA receptor antagonist (Fig 8). The increased excitability of the 5-HTergic neural circuits in the DRN by NaSal may lead to a raised 5-HT level in the rodent brain.

### NaSal targets GABAergic neurons in the DRN

GABAergic neurons are abundant throughout the DRN [10,18,47] and most of them are inhibitory interneurons, which form synaptic contacts with 5-HTergic cell bodies or dendrites [48,49,50,51,52]. In the rat, we used the fast-spiking [19] as one of critical criteria for identifying GABAergic neurons (Fig 1C and S1 Fig). In the ChR2 transgenic mouse, we mainly relied on the excitatory response to blue light for identifying GABAergic neurons (Fig 6A). In both the rat and the mouse, those neurons were sensitive to NaSal in terms of the suppressed functional response (Figs 2–4 and 7).

NaSal hyperpolarized the resting membrane potential, decreased the input resistance and suppressed action potential firing in GABAergic neurons (Figs 2–4 and 7), indicating that the drug decreases the excitability of those neurons. Although how NaSal lowers the excitability of GABAergic neurons in the DRN has not been known, we suspect that the drug likely targets the membrane ion channels because it hyperpolarized the resting membrane potential (Figs 2 and 7F). Because it decreased the input resistance (Figs 2 and 7F), we further suspect that it opens some ion channels to allow an influx of anions or an outflow of cations. Since NaSal attenuates the current mediated by glycine receptors [53] or GABA_A_ receptors [54,55], it does not likely activate chloride channels. NaSal possibly potentiates efflux of potassium ions from the cell by opening the potassium ion channels associated with resting membrane potential homeostasis. Indeed, our recent study [56] shows that NaSal targets metabotropic GABA_B_ receptors to activate G protein-gated inwardly-rectifying potassium channels (GIRKs) [57,58], which hyperpolarizes the resting membrane potential and decreases the input resistance in neurons of rat medial geniculate body [56]. Further investigation is required to elucidate the mechanism underlying the process in which NaSal lowers the excitability of GABAergic neurons in the DRN.

NaSal suppressed the frequency and the amplitude of GABAergic sIPSCs in 5-HTergic neurons (Figs 5A and 9; Table 3), indicating that the drug impairs the GABAergic synaptic transmission. Further more, NaSal reduced the frequency, but not the amplitude, of mIPSCs in 5-HTergic neurons (Fig 5B; Table 3), suggesting that the drug impairs the GABAergic synaptic transmission through a presynaptic mechanism. These findings are similar to those we previously found in the inferior colliculus [24] and the auditory cortex [59]. NaSal attenuates the GABAergic synaptic transmission to 5-HTergic neurons likely by suppressing action potential firing of presynaptic GABAergic neurons and probably by blocking voltage-sensitive Ca^2+^ channels [60] on the presynaptic membrane to reduce GABA release. Further study is required to elucidate the presynaptic signaling pathways in which NaSal suppresses the GABAergic synaptic transmission.

### NaSal raises the excitability of 5-HTergic neurons in the DRN

NaSal increased the current-evoked firing of 5-HTergic neurons with enhanced GABAergic inhibition by optical stimulation to the DRN of the ChR2 transgenic mouse (Fig 7A), indicating that the drug can raise the excitability of 5-HTergic neurons. We believe that NaSal increases the excitability at least by targeting the GABAergic neurons, because the drug would fail to do so when the GABAergic synaptic transmission was blocked by PTX, a GABA receptor antagonist (Fig 8). In other words, NaSal releases 5-HTergic neurons from the GABAergic inhibition by lowering the excitability of GABAergic neurons and by attenuating the GABAergic transmission through a presynaptic mechanism. NaSal does not likely increase the excitability of 5-HTergic neurons by targeting postsynaptic GABA receptors because the drug did not significantly change the amplitude of mIPSCs in those neurons (Fig 5B; Table 3). NaSal does not likely increase the excitability of 5-HTergic neurons by directly targeting those neurons because the drug did not increase their firing evoked by a large depolarizing current ≥40 pA (Fig 7A), in which case the effects of presynaptic inputs are minimized [61].

GABAergic fast-firing interneurons in the local DRN provide a negative feedback to the 5-HTergic output [10,18,19]. The suppressed activity of these neurons by NaSal (Figs 2–4) should have increased the excitability of 5-HTergic neurons in rat DRN, but this did not occur (Figs 2–4). We speculate that a certain baseline of the GABAergic inhibition is a prerequisite for NaSal to significantly disinhibit 5-HTergic neurons. In brain slices, however, the floor of the GABAergic inhibition is too low because many of GABAergic projections onto 5-HTergic neurons are severed and GABAergic neurons are less spontaneously active than those in the *in vivo* condition. As a matter of fact, the mean spontaneous discharge rate of GABAergic neurons recorded in the present study was ~3.5 Hz (Fig 3B, right panel), in contrast to a high rate up to 12 Hz as observed *in vivo* [19]. To raise the floor of the GABAergic inhibition to 5-HTergic neurons, we optically excited GABAergic neurons with a blue laser light (473 nm) in DRN slices of the ChR2 transgenic mouse. It is true that the increased excitability of 5-HTergic neurons by NaSal was successfully manifested (Fig 7A) in the DRN of the ChR2 transgenic mouse after GABAergic inhibition was enhanced by optical stimulation (Fig 6B).

In the rat, there exist so called glutamic acid decarboxylase (GAD) 67-expressing 5-HTergic neurons (5-HT/GAD67 neurons), which release and synthesize GABA [39]. However, the neurons coexpressing 5-HT and GAD67 considerably decrease in population between 4–8 weeks old in the rat DRN [39]. Thus, we believe that there are few 5-HT/GAD neurons in the ChR2 transgenic mouse used in our study which was 4–6 weeks old. Moreover, coexistence of 5-HT/tryptophan hydroxylase 2 (TPH2) and GABA/GAD is absent or very rare in the rat DRN [33,62]. Accordingly, we think that activation of 5-HT/GAD67 neurons by optical stimulation has minimal influence on 5-HTergic neurons in the ChR2 transgenic mouse.

### Direct pharmacological action of NaSal on the DRN at least partially contributes to the raised 5-HT level in the brain

NaSal, a tinnitus inducing agent, can activate 5-HTergic neurons in the DRN [27] and can increase 5-HT level in the inferior colliculus and the auditory cortex [23] in rodents. One mechanism for NaSal-induced elevation in the 5-HT level may underlie the stress imposed by NaSal-induced tinnitus. It has been reported that the auditory stressor, such as the noise exposure, could increase 5-HT levels in discrete regions of the rat brain [63]. Thus, it is possible that NaSal-induced tinnitus indirectly activates the 5-HTergic neural circuits in the rodent brain. The findings in the present study raise another possibility: 5-HTergic neurons in the DRN are activated by direct pharmacological actions of NaSal on the GABAergic neurons, which contributes to the raised 5-TH level in the brain.

Early studies show that the central auditory nuclei receive rich 5-HTergic fibers and terminal endings originating from the raphe nuclei. Dense 5-HT_2_ and 5-HT_1A_ receptors are found in the primary auditory cortex [64] and 5-HT_1A_ receptors in the cochlear nucleus and the inferior colliculus. The network of 5-HTergic innervation from the raphe nuclei is likely active primarily in the modulation of sound perception [14]. Much evidence indicates that tinnitus is closely related to 5-HT functions [65,66,67,68,69]. The present study shows that NaSal raises the excitability of 5-HTergic circuits in the DRN, which may help us to understand the relationship between tinnitus and the 5-HTergic system.

## Conclusions

In the present study, we show that NaSal preferentially suppresses the GABAergic inhibitory activity to increase the excitability of 5-HTergic neurons in the DRN, which may contribute to the raised 5-HT level in the brain of rodent animals by NaSal.

## Supporting Information

S1 Fig Distinct properties of the current-evoked firing between identified 5-HTergic and GABAergic neurons of rat dorsal raphe nucleus (DRN). Input—output functions (A), inter-spike intervals (B) and action potential morphology (C) of current-evoked firing. Sample sizes are indicated in inset. Vertical line bars represent one standard error. *P <0.05; **P <0.01 (unpaired Student’s *t*-test).
(PDF)

S2 Fig Time constant (tau) of current-voltage responses was distinct between identified 5-HTergic and GABAergic neurons of rat DRN. The tau is defined as the time required to reach 63% of the maximum magnitude of the voltage response. (A) Sample current-voltage responses to a series of current steps (500 ms duration, -30 pA to -80 pA, -10 pA/step) recorded from a 5-HTergic neuron and from a GABAergic neuron. Blow up of the membrane potential responses (indicated by asterisks) within a time window of tau to a -30 pA current pulse is shown. Arrows indicate onset of the current pulse. Dashed lines are exponential functions fit to the responses. (B) Scatter plots of tau for two types of neurons. (C) Bar graph showing mean tau for 5-HTergic neurons (n = 14) was longer than that for GABAergic neurons (n = 11). Vertical line bars represent one standard error. *P <0.05 (unpaired Student’s *t*-test).
(PDF)

S3 Fig Distinct pharmacological properties between identified 5-HTergic and GABAergic neurons of rat DRN. (A) Sample membrane current traces (left panels) and scatter plots (right panels) showing that application of 100 μM 5-HT evoked an outward current in 5-HTergic, but an inward current in GABAergic, neurons. Application of 5 μM 8-OH-DPAT, a 5-HT_1A_ receptor agonist, evoked an outward current in 5-HTergic neurons, but evoked a minimal current in GABAergic neurons. Vertical line bars represent one standard error. (B) Sample raw traces showing that 100 μM 5-HT suppressed action potential firing induced by phenylephrine (PE, 3 μM) in 5-HTergic neurons, but increased spontaneous action potential firing in GABAergic neurons. Note that PE-induced firing in 5-HTergic neurons could be blocked by 5 μM 8-OH-DPAT.
(PDF)

S4 Fig Sodium salicylate (NaSal) suppressed current-evoked firing in GABAergic, but not in 5-HTergic, neurons, of rat DRN. The graphs show typical patterns of firing rate in response to a series of current steps (0 to 80 pA re threshold, 10 pA/step) recorded from a 5-HTergic neuron and a GABAergic neuron. Solid horizontal bars indicate time course of NaSal application.
(PDF)

S5 Fig NaSal increased the threshold of current-evoked firing in GABAergic, but not in 5-HTergic, neurons of rat DRN. Sample traces (A) and statistics (B) showing that NaSal increased the threshold current for evoking an action potential in GABAergic (n = 7), but not in 5-HTergic (n = 8), neurons. Vertical line bars represent one standard error. **P <0.01 and ^NS^ *P* >0.05 relative to control (two-way RM-ANOVA and paired Student’s *t*-test).
(PDF)

S6 Fig Spontaneous inhibitory postsynaptic responses were mediated by GABA receptors in both 5-HTergic and GABAergic neurons of rat DRN. Sample traces showing that spontaneous inhibitory postsynaptic currents (sIPSCs) recorded in a 5-HTergic neuron could be reversibly inhibited by 10 μM bicuculline (BIC), a selective GABA_A_ receptor antagonist.
(PDF)
