# Supplementary figures and images for: Sodium Salicylate Suppresses GABAergic Inhibitory Activity in Neurons of Rodent Dorsal Raphe Nucleus
Source: PLoS One. 2015 May 11;10(5):e0126956. doi: 10.1371/journal.pone.0126956 (PMC4427486; doi:10.1371/journal.pone.0126956)

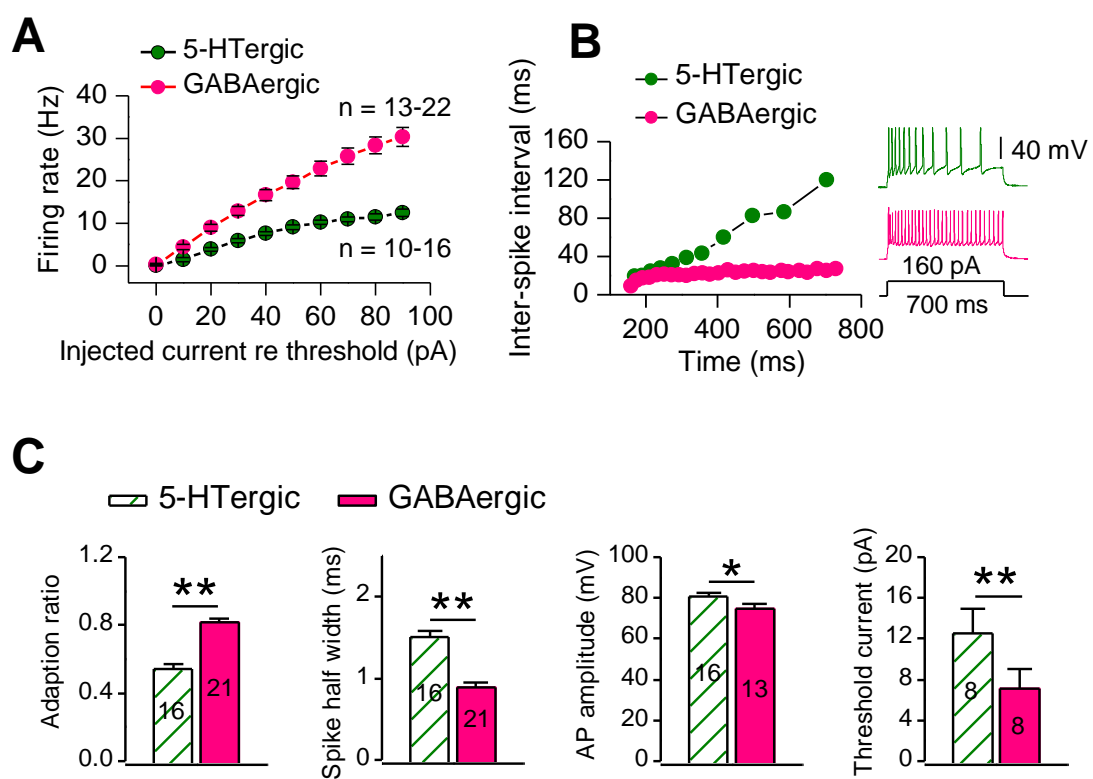

Supplement: S1 Fig — Input—output functions (A), inter-spike intervals (B) and action potential morphology (C) of current-evoked firing. Sample sizes are indicated in inset. Vertical line bars represent one standard error. *P <0.05; **P <0.01 (unpaired Student’s t-test). (PDF) [file pone.0126956.s001.pdf]

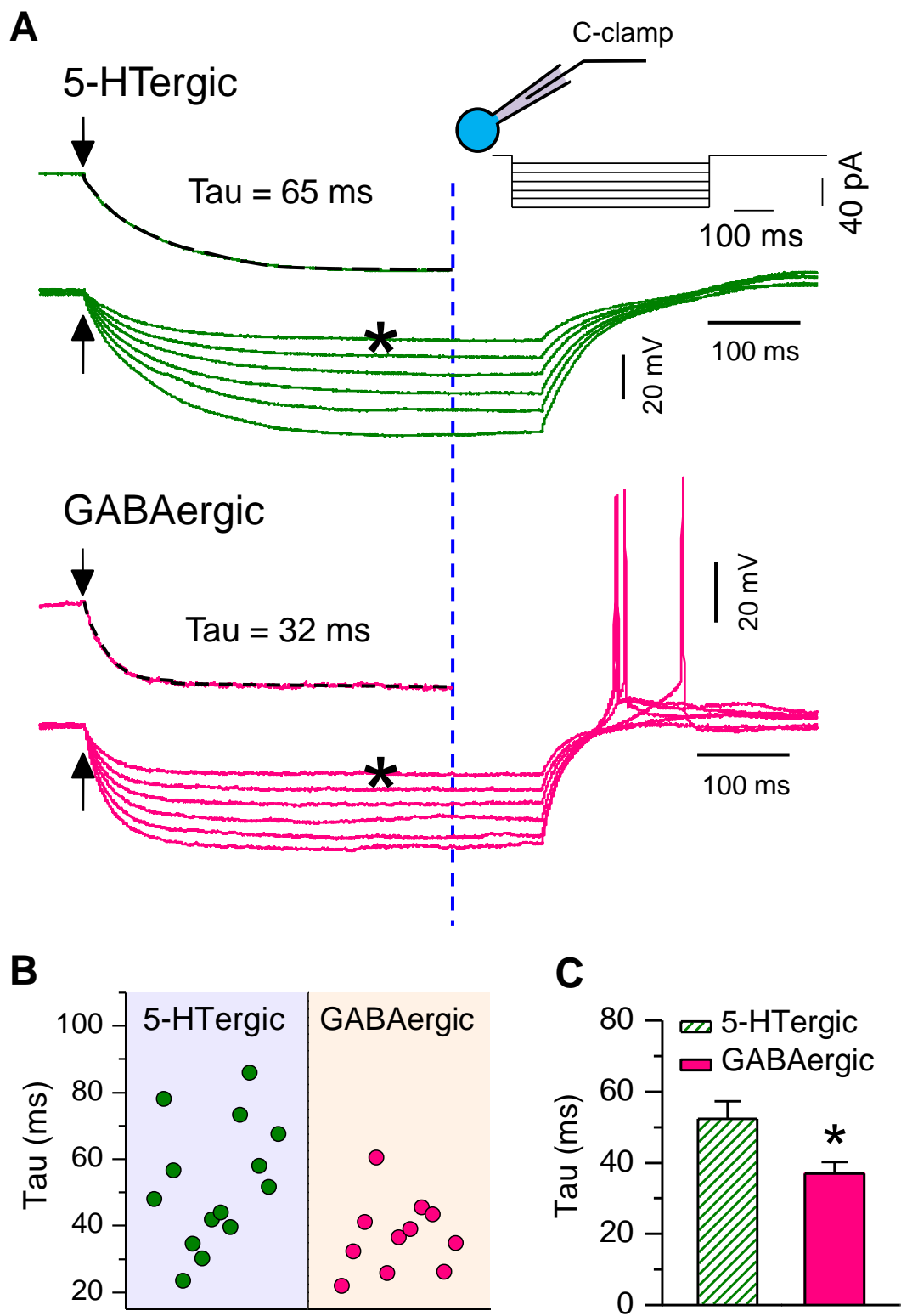

Supplement: S2 Fig — The tau is defined as the time required to reach 63% of the maximum magnitude of the voltage response. (A) Sample current-voltage responses to a series of current steps (500 ms duration, -30 pA to -80 pA, -10 pA/step) recorded from a 5-HTergic neuron and from a GABAergic neuron. Blow up of the membrane potential responses (indicated by asterisks) within a time window of tau to a -30 pA current pulse is shown. Arrows indicate onset of the current pulse. Dashed lines are exponential functions fit to the responses. (B) Scatter plots of tau for two types of neurons. (C) Bar graph showing mean tau for 5-HTergic neurons (n = 14) was longer than that for GABAergic neurons (n = 11). Vertical line bars represent one standard error. *P <0.05 (unpaired Student’s t-test). (PDF) [file pone.0126956.s002.pdf]

**S3 Figure**

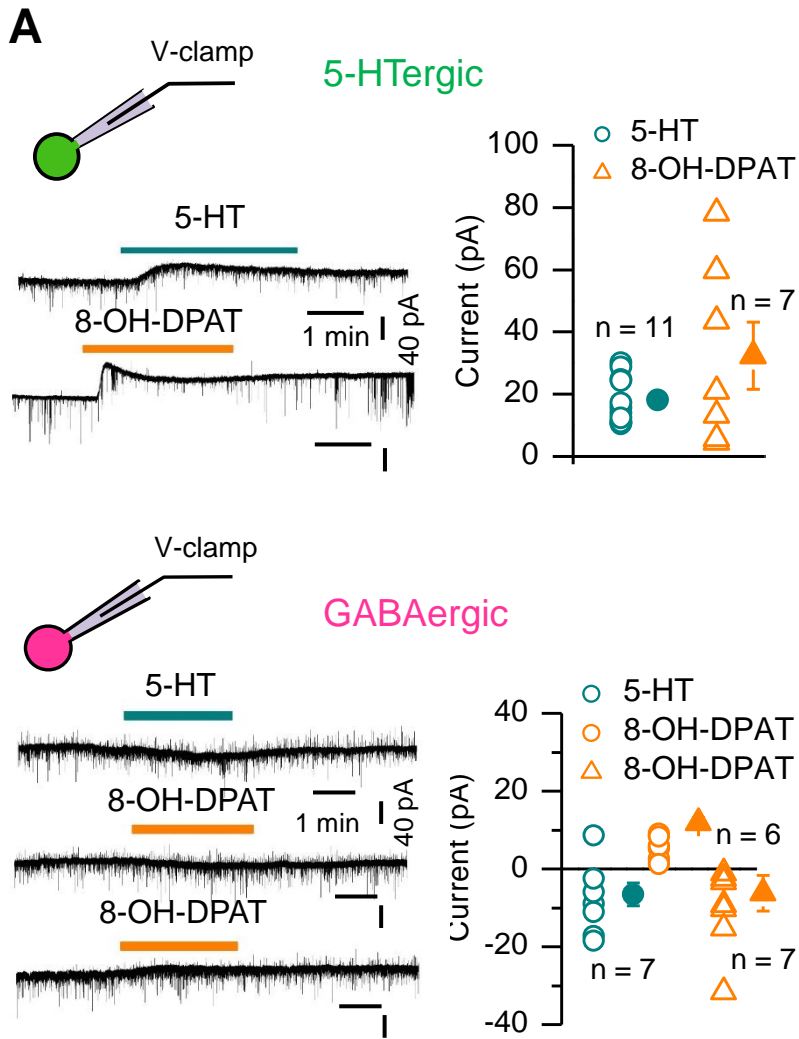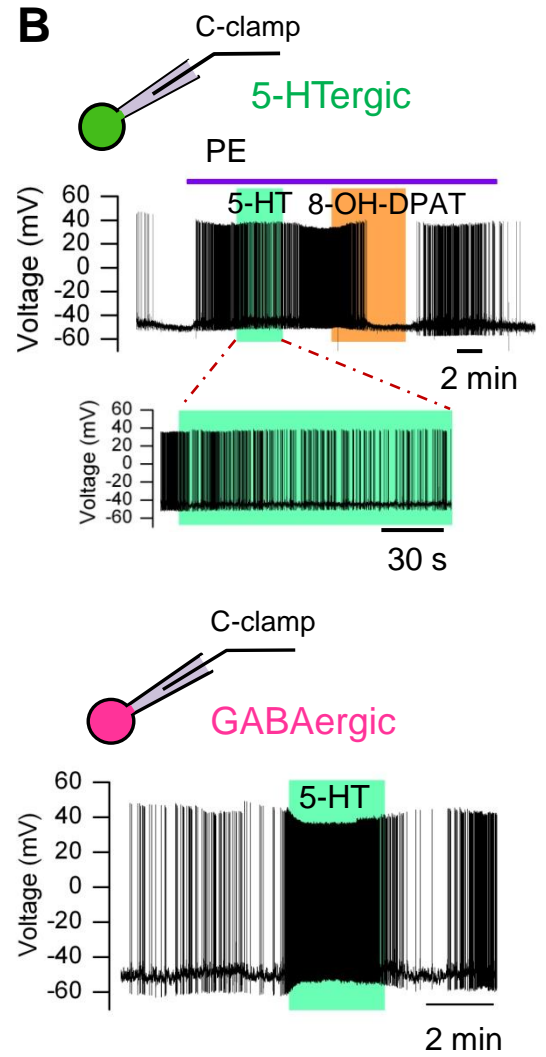

Supplement: S3 Fig — (A) Sample membrane current traces (left panels) and scatter plots (right panels) showing that application of 100 μM 5-HT evoked an outward current in 5-HTergic, but an inward current in GABAergic, neurons. Application of 5 μM 8-OH-DPAT, a 5-HT1A receptor agonist, evoked an outward current in 5-HTergic neurons, but evoked a minimal current in GABAergic neurons. Vertical line bars represent one standard error. (B) Sample raw traces showing that 100 μM 5-HT suppressed action potential firing induced by phenylephrine (PE, 3 μM) in 5-HTergic neurons, but increased spontaneous action potential firing in GABAergic neurons. Note that PE-induced firing in 5-HTergic neurons could be blocked by 5 μM 8-OH-DPAT. (PDF) [file pone.0126956.s003.pdf]

## S4 Figure

5-HTergic

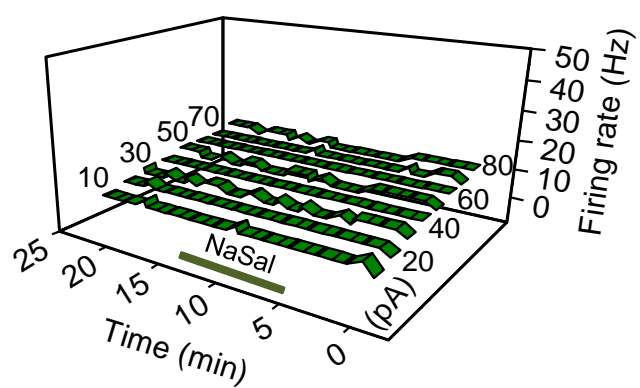

GABAergic

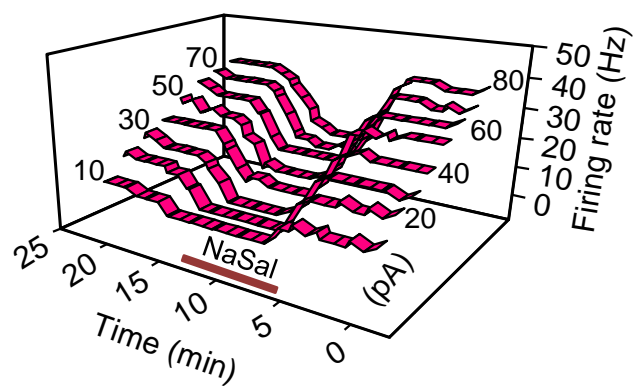

Supplement: S4 Fig — The graphs show typical patterns of firing rate in response to a series of current steps (0 to 80 pA re threshold, 10 pA/step) recorded from a 5-HTergic neuron and a GABAergic neuron. Solid horizontal bars indicate time course of NaSal application. (PDF) [file pone.0126956.s004.pdf]

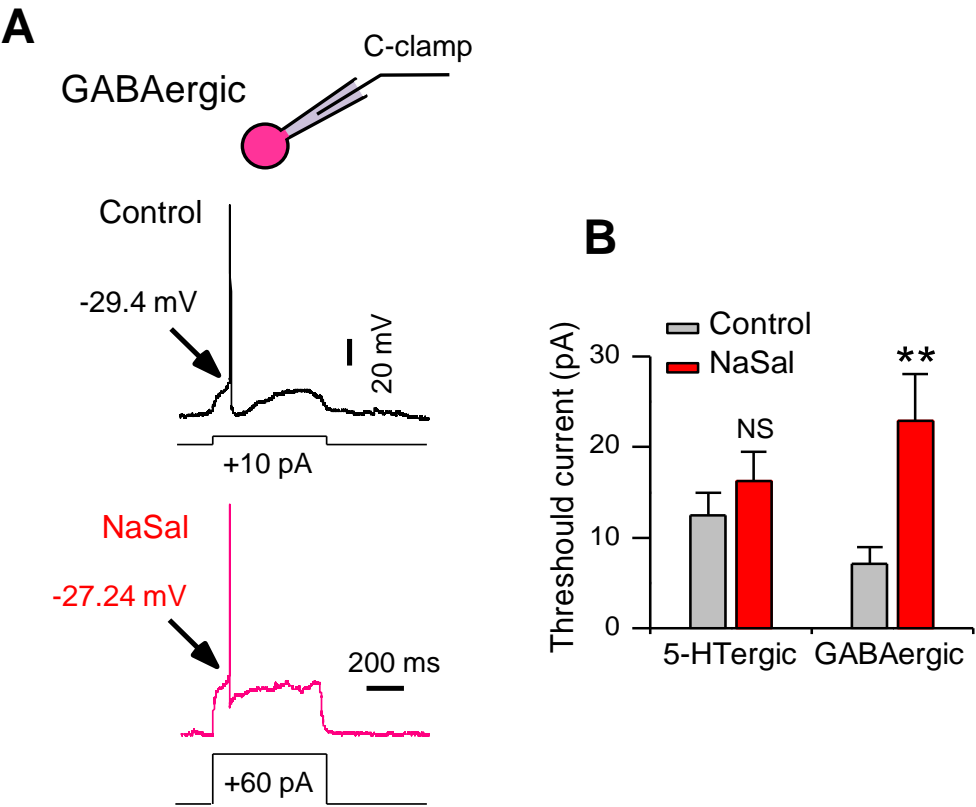

Supplement: S5 Fig — Sample traces (A) and statistics (B) showing that NaSal increased the threshold current for evoking an action potential in GABAergic (n = 7), but not in 5-HTergic (n = 8), neurons. Vertical line bars represent one standard error. **P <0.01 and NS P >0.05 relative to control (two-way RM-ANOVA and paired Student’s t-test). (PDF) [file pone.0126956.s005.pdf]

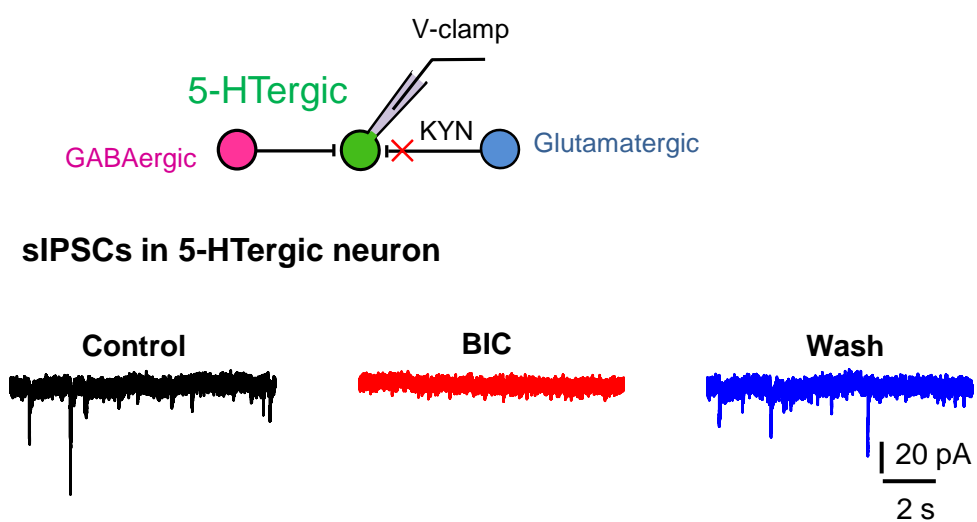

Supplement: S6 Fig — Sample traces showing that spontaneous inhibitory postsynaptic currents (sIPSCs) recorded in a 5-HTergic neuron could be reversibly inhibited by 10 μM bicuculline (BIC), a selective GABAA receptor antagonist. (PDF) [file pone.0126956.s006.pdf]
